# Maternal and Perinatal Outcome in Women with Congenital Heart Disease: An Observational Study

**DOI:** 10.31729/jnma.9015

**Published:** 2025-05-31

**Authors:** Pooja Paudyal, Asmita Ghimire, Bashu Dev Parajuli, Prabhat Khakural

**Affiliations:** 1 Department of Obstetrics and Gynaecology, Tribhuvan University Teaching Hospital, Institute of Medicine, Maharajgunj, Kathmandu, Nepal; 2 Department of Anaesthesiology, Tribhuvan University Teaching Hospital, Institute of Medicine, Maharajgunj, Kathmandu, Nepal; 3 Department of Cardiothoracic and Vascular Surgery, Manmohan Cardiothoracic Vascular and Transplant Center, Institute of Medicine, Maharajgunj, Kathmandu, Nepal

**Keywords:** *congenital heart disease*, *Eisenmenger syndrome*, *fetal outcomes*, *pregnancy outcomes*

## Abstract

**Introduction:**

Untreated and residual congenital heart disease in a pregnant woman is concerning for both the mother and the baby. Early diagnosis and management are imperative to ensure survival of both mother and the baby. The aim was to study the maternal and perinatal outcomes in women with congenital heart disease.

**Methods:**

An observational study was conducted in a tertiary care hospital over a three-year period from April 2020 to March 2023. The data was collected retrospectively after ethical approval from the Institutional Review Committee [Reference number: 373/ (6-11) E2/076/077]. Total sampling was done where all women with congenital heart disease who delivered in the hospital after twenty-eight weeks of gestation during the study period were enrolled. Data were collected from the record book of labour room, patient files, and labour room, along with mortality audits of the department. Maternal and fetal outcomes were recorded, and descriptive analyses were done.

**Results:**

Seventy-three women with congenital heart disease delivered in our center during the study period. The average age of the women was 26.51+ 5.50 years. Among them, 39 (53.4%) of the patients had uncorrected heart conditions. Atrial septal defect was present in 20 (27.39%) pregnant patients. There were 69 (94.52%) live births and four (5.47%) intrauterine foetal deaths. One (1.36%) newborn was diagnosed to have an ostium secundum atrial septal defect, and two (2.73%) newborns were diagnosed to have patent foramen ovale.

**Conclusions:**

The maternal and perinatal outcomes in women with treated congenital heart disease are favourable, whereas the maternal mortality remains high in women with Eisenmenger Syndrome.

## INTRODUCTION

Congenital heart disease (CHD) is seen in 0.8% to 1.2% of live births worldwide.^1,2^ Heart diseases complicate 1% to 4% of pregnancies and account for 10% to 15% of maternal mortality.^3,4^ With improvement in cardiac surgery outcomes, the incidence of pregnant women with CHD is increasing.^5^ The previous ratio of 3:1 for rheumatic heart disease to CHD is now reversed in developed countries.^6,7^ There is an increased number of young women with CHD reaching the childbearing age.^8^ Such pregnancies are at higher risk of foetal and neonatal complications.^9^

There are not many studies on the feto-maternal outcome in women with CHD from Nepal. The study site being a tertiary referral center with availability of both cardiac and obstetric services, cases get referred from all over the country.

This study was done to study the maternal and fetal outcomes in patients with CHD at our center.

## METHODS

This observational study was conducted in the Department of Obstetrics and Gynaecology of a tertiary care center in Kathmandu, Nepal. The duration of the study was three years from April 2020 to March 2023. Ethical clearance was obtained from the Institutional Review Committee of the Institute of Medicine [Reference number: 373/ (6-11) E2/076/077]. Total sampling was done. All women with congenital heart disease who delivered in the hospital after twenty-eight weeks of gestation during the study period were enrolled. Women with previously diagnosed and treated congenital heart disease were included as well as women first diagnosed with CHD during pregnancy. The diagnosis was made according to the findings of echocardiography done by a consultant cardiologist. Women with CHD who delivered before twenty-eight weeks of gestation were excluded. Pregnant women with other types of heart disease apart from CHD, such as ischemic heart disease, rheumatic heart disease, cardiomyopathies, and those with valve replacement, were not included in the study.

Data were collected from the record book of the labour room, patient files, and the labour room, along with mortality audits of the department. For this study, data were collected regarding age, gravidity, parity of the patients, details of heart disease-New York Heart Association functional class, type of congenital heart disease, surgery done, and feto-maternal outcome. For maternal outcome-heart failure, arrhythmia, stroke, myocardial infarction, and mortality were the study parameters, and for the fetal outcome - intrauterine fetal death, low birth weight, low APGAR scores, neonatal intensive care unit admissions, and neonatal deaths were recorded.

All data were entered into a master chart, and descriptive analyses in the form of numbers, percentages, and means were calculated using the IBM SPSS Statistics version 21 (IBM Corp., Armonk, N.Y., USA).

## RESULTS

There were a total of 73 women with congenital heart disease included in this study who delivered beyond 28 weeks of gestation in our center during the study period of three years. The average age of the women was 26.51 ± 5.50 (range, 17 years - 41 years) years, with 51 (69.86%) women belonging to the 20-29 years age group. Out of the total, 40 (54.79%) were primigravida, and three (4.10%) women were grand multipara, having given birth five times or more (Table 1).

**Table 1:** Characteristics heart disease (n=73).

Characteristics	n (%)
Age group (years)	
<20	1 (1.36)
20-29	51 (69.86)
30-39	18 (24.65)
40-49	3 (4.10)
Gravidity	
Primigravida	40 (54.79)
Multigravida	30 (41.09)
Grand multigravida	3 (4.10)

Atrial septal defect, ventricular septal defect, and tetralogy of Fallot were the top three presenting congenital cardiac lesions in 41 (56.16%), 17 (23.28%), and seven (9.58%) patients, respectively (Table 2). Among all the women, 34 (46.57%) had undergone surgery for congenital cardiac lesions before pregnancy, while 39 (53.42%) had entered pregnancy without surgical correction of their lesions. Five women (6.84%) with uncorrected ventricular septal defects had developed Eisenmenger Syndrome.

**Table 2:** Details of heart disease (n = 73).

Disease	Corrected n (%)	Uncorrected n (%)	Total n (%)
Atrial septal defect	20 (27.39)	19 (26.02)	39 (53.42)
Atrial septal defect with moderate pulmonary artery hypertension	-	1 (1.36)	1 (1.36)
Atrial septal defect with severe pulmonary stenosis	-	1 (1.36)	1 (1.36)
Ventricular septal defect	3 (4.10)	9 (12.32)	12 (16.43)
Ventricular septal defect with Eisenmenger syndrome	-	5 (6.84)	5 (6.84)
Arial septal defect and ventricular septal defect	-	1 (1.36)	1 (1.36)
Tetralogy of Fallot	7 (9.58)	-	7 (9.58)
Isolated pulmonary stenosis	3 (4.10)	1 (1.36)	4 (5.47)
Moderate pulmonary stenosis	3 (4.10)	-	3 (4.10)
Severe pulmonary stenosis	-	1 (1.36)	1 (1.36)
Atrioventricular canal defect	1 (1.36)	-	1 (1.36%)
Coarctation of aorta	-	1 (1.36)	1 (1.36)
Patent ductus arteriosus with mild mitral regurgitation	-	1 (1.36)	1 (1.36)
Total	34 (46.57)	39 (53.42)	73 (100)

**Table 3:** Mode of delivery (n=73).

Mode of delivery	n (%)
Normal vaginal delivery	31 (42.46)
Instrument vaginal delivery	5 (6.84)
Caesarean section	37 (50.68)
Elective caesarean section	12 (16.43)
Emergency caesarean section	25 (34.24)
Indication of caesarean section	
Obstetric indication	30 (41.09)
Severe pulmonary artery hypertension	2 (2.73)
Congestive heart failure	2 (2.73)
Eisenmenger syndrome	3 (4.10)

**Table 4:** Maternal complications.

Complications	n (%)
Heart failure	6 (8.21)
Supra-ventricular tachycardia	1 (1.36)
Mortality	2 (2.73)

**Table 5:** Fetal complications (n=42).

Complications	n (%)
Low birth weight	14 (19.17)
Preterm	14 (19.17)
Low APGAR	8 (10.95)
Intrauterine fetal death	4 (5.47)
Neonatal death	2 (2.73)

Thirty-seven women (50.68%) underwent lower segment caesarean section, out of which 30 (41.09%) were for obstetric indications and seven (9.58%) were for cardiac reasons. Among obstetric indications, fetal distress was the most common cause for Caesarean section (CS). In seven (18.91%) women, caesarean section had to be performed primarily for cardiac reasons, as vaginal delivery was not feasible due to their cardiac status, as they had severe pulmonary arterial hypertension in two (5.40%), congestive cardiac failure prior to CS in two (5.40%) and three (8.10%) had Eisenmenger Syndrome. Thirty-six women (49.31%) had vaginal deliveries, among which five (6.84%) were instrumental (Table 3).

Among total, 67 (91.73%) required care in the high dependency unit during the peripartum period. Nine (12.33%) patients had cardiac complications-six (8.21%) developed heart failure, one (1.73%) had supraventricular tachycardia, and there were two (2.73%) mortalities, both in women with Eisenmenger syndrome (Table 4). None of the women included in our study had a stroke or myocardial infarction.

Among the 73 pregnant women, there were 69 (94.52%) live births and four (5.47%) intra-uterine foetal deaths (Table 5). One intra-uterine fetal death was due to abruptio-placenta at 36 weeks in a woman with Ventricular Septal Defect, one in a woman with coarctation of the aorta at 29 weeks and the other two intra-uterine foetal deaths were in term pregnancy in women with Atrial septal defect without any obvious reasons. Fourteen (19.17%) babies were delivered preterm, while 59 (80.82%) reached term. Fourteen (19.17%) babies were small for gestational age, while the rest were appropriate for gestational age. The birth weight ranged from 0.77-4 kilograms, with an average being 2.741 ± 0.587 kilograms. Prematurity was present in 14 (19.17%) babies. Low APGAR score was seen in 8 (10.9%) babies at one and five minutes of life, and they needed resuscitation. Ten (14.49%) babies needed admission to the neonatal unit, and five (7.25%) were admitted to the neonatal intensive care unit. There were two (2.89%) early neonatal deaths among the 69 live-born babies (Table 5).

## DISCUSSION

With the advancements in management of congenital heart disease, even those with complex lesions can attain adulthood and a chance at motherhood. Normal physiological changes to the heart and blood vessels during pregnancy cause increased workload on the heart. Especially in a pregnant woman with congenital cardiac disease, cardiac decompensation can occur as a result of increases in cardiac output, blood volume, contractility, and heart rate can cause a significant circulatory burden.^4^ This can lead to complications in both mother and baby, but with timely interventions, these complications are preventable, leading to improved feto-maternal outcome.

In the present study, the average age of the women was 26.51 ±5.50 years, with 51 (69.86%) women belonging to the 20-29 years age group. 40 (54.79%) of the women were primigravida, with many opting to have more babies. Similar to our finding, in a study by Ezzeldin et.al, the mean age of the patients was 35.4 ± 12.2 years, and the mean age at time of first pregnancy was 22.77 ± 2.97 years. Contrary to our study, 97.7% of these patients were multigravida.^7^

The commonest congenital heart disease in our pregnant patients was an atrial septal defect, which is a finding similar to studies from Egypt and Spain.^7^-^9^ Thomsons et al also reported atrial septal defects (22.6%) as the prevalent lesion, followed by ventricular septal defects (14.5%).^4^ However, the majority of the patients in our study had uncorrected cardiac lesions. The reasons behind this could be a lack of cardiac screening and non-diagnosis, delayed diagnosis, difficult geography, and inaccessibility to healthcare facilities. Even patients with Eisenmenger syndrome were coming to us with pregnancy, against the long-established guideline of avoiding pregnancy in Eisenmenger Syndrome. It is again because of the ignorance and family pressure in our society.

The number of patients presenting to us in New York Heart Association Class I, II, III, and IV was sixty-seven, one, four, and one, respectively. Uncorrected lesions presented with New York Heart Association classes III and IV.

Peri-partum heart failure was the commonest complication seen in six patients, and one patient had supraventricular tachycardia requiring medical management. In a study of 562 pregnant women with heart disease receiving care in 13 Canadian cardiac and obstetric teaching hospitals, Siu et. al, found that composite cardiovascular outcome (pulmonary oedema, arrhythmia, stroke, or cardiac death) complicated 13% of 617 pregnancies.^11^ Furenas and his colleagues reported less incidence of cardiac complications in pregnant women with congenital heart diseases at a rate of 14%, however arrhythmia was the commonest complication (6%) followed by heart failure in five patients.^12^ A study by Ezzeldin et al had considerable maternal morbidity with 52.2 % developing complications including heart failure, arrythmia and infective endocarditis.^7^ Similarly, Thompsons et al. reported that among all 655 women with CHD, the incidence of cardiovascular complications was 4.1%, mostly due to arrhythmia, heart failure, stroke, and myocardial infarction.^4^

Maternal mortality was seen in 2 (2.73%) patients in our study, and both occurred in the postpartum period. Manso et. al, also reported 2 deaths out of 56 patients during the postpartum period, while no mortality was seen in the study by Ezzeldin.^9,12^ Those two patients in our study had a ventricular septal defect with Eisenmenger Syndrome. Maternal mortality in pregnant women with Eisenmenger Syndrome is high. Various studies report it to be ranging from 30 to 50% and increasing to 65% in patients undergoing caesarean section.^13-15^ We also had 40% mortality in such patients; only three out of the five cases of Eisenmenger Syndrome could be saved. Pregnancy with Eisenmenger Syndrome has also been reported by Pandey et. al, from India, where they managed three cases of pregnancy with Eisenmenger Syndrome with good outcome.^10^ Earlier diagnosis and surgical correction of the Ventricular septal defect could have prevented the development of Eisenmenger Syndrome in these women and offered them a favourable outcome. Inaccessibility to health care facilities, financial issues, and geographical barriers could have led to a delayed diagnosis of congenital heart disease in our setting.

All of our patients had a singleton pregnancy. In a study by Manso et. al, four twin pregnancies were also included.^9^ The mode of delivery was normal vaginal delivery, instrument vaginal delivery and caesarean section in 31 (42.46%), 5 (6.84%), 37 (50.68%) patients, respectively. Manso et al reported higher vaginal and instrumental deliveries as compared to our study (vaginal delivery 50.74%, instrumental 22.38% and CS 26.86%).^9^

There were 69 live births and four intrauterine foetal deaths in our study. Out of the total 73 deliveries, 14 (19.17%) were delivered preterm, while in a study by Manso et.al, there were also 69 live births, out of which ten were premature births.^9^ Our study had a higher proportion of 19.17% of the babies were small for gestational age 14 (19.17%) whereas only one had a low birth for the gestational age in the study by Manso et. al.^9^ The average birth weight 2700 grams and range of birth weight of 770-4000 grams was similar as in the other study, weight 2830 grams (range, 1000 4250 grams).^9^ In our study, one (1.36%) newborn was diagnosed to have an Ostium Secundum Atrial Septal Defect, and two (2.73%) newborns were diagnosed to have Patent Foramen Ovale. Ezzeldin et. al, found that 12 (13.4%) out of 90 patients had offspring with congenital heart disease; the most common inherited defect was Atrial Septal Defect as seen in 9.0%.^7^ In our center, there were five (6.84%) neonatal intensive care unit admissions and two (2.73%) neonatal deaths. Both neonatal deaths occurred in women with Eisenmenger Syndrome in whom the pregnancies were electively terminated at 28 and 29 weeks, respectively, due to worsening maternal condition. The babies weighed 770 grams and 900 grams, respectively, and died due to complications of prematurity, mainly respiratory distress syndrome of prematurity.

As more and more women with congenital cardiac diseases reach childbearing age, these women form a distinct group who need specialized care during their pregnancies. Starting from pre-conceptional optimization of their cardiac status to providing a focused antenatal care and safe delivery, their management entails the efforts of obstetricians, cardiologists, cardiac surgeons, anaesthetists, and neonatologists in a specialized center. Those with corrected lesions have a fairly good outcome. With efforts from a dedicated multidisciplinary team, these high-risk women can be helped in their quest for motherhood.

Our study was a single-center, retrospective, descriptive study of three years duration only. The sample size was small. Pregnant women delivering before twenty-eight weeks of gestation were not studied. Sub-group analysis of feto-maternal outcome in repaired and unrepaired congenital heart disease was not carried out.

## CONCLUSION

Our study demonstrated a good feto-maternal outcome in women with congenital heart disease with optimum medical care. Both maternal and neonatal mortality occurred in women with Eisenmenger Syndrome, highlighting the need for timely screening, diagnosis, and intervention in patients with congenital cardiac lesions.

## References

[1] Bouma BJ, Mulder BJ (2017). Changing Landscape of Congenital Heart Disease.. Circ Res..

[2] van der Linde D, Konings EE, Slager MA, Witsenburg M, Helbing WA, Takkenberg JJ (2011). Birth prevalence of congenital heart disease worldwide: a systematic review and meta-analysis.. J Am Coll Cardiol..

[3] Pushpalatha K (2010). Cardiac diseases in pregnancy-A review.. JIMSA..

[4] Thompson JL, Kuklina EV, Bateman BT, Callaghan WM, James AH, Grotegut CA (2015). Medical and Obstetric Outcomes Among Pregnant Women with Congenital Heart Disease.. Obstet Gynecol..

[5] Greutmann M, Pieper PG (2015). Pregnancy in women with congenital heart disease.. Eur Heart J..

[6] Caruana M, Gatt M, Aquilina O, Ventura CS, Grech V, Somerville J (2023). The impact of maternal congenital heart disease on pregnancy outcomes in Malta: a retrospective study.. Global Cardiology..

[7] Ezzeldin DA, Roshdy A, Attia H, Elfiky A, Nour A (2017). Pregnancy in Adult Congenital Heart Disease Maternal and Fetal Outcome, a Single Centre Study in a Developing Country.. J Cardiovasc Dis Diagn..

[8] Lu CW, Shih JC, Chen SY, Chiu HH, Wang JK, Chen CA (2015). Comparison of 3 Risk Estimation Methods for Predicting Cardiac Outcomes in Pregnant Women with Congenital Heart Disease.. Circ J..

[9] Manso B, Gran F, Pijuan A, Giralt G, Ferrer Q, Betrian P (2008). Embarazo y cardiopatias congenitas [Pregnancy and congenital heart disease].. Rev Esp Cardiol..

[10] Pandey D, Suri J, Bharti R, Mittal P (2024). Pregnancy Outcome in Eisenmenger Syndrome at an Indian Tertiary Center.. J South Asian Feder Obst Gynae.

[11] Siu SC, Sermer M, Colman JM, Alvarez AN, Mercier LA, Morton BC (2001). Prospective multicenter study of pregnancy outcomes in women with heart disease.. Circulation..

[12] Furenas E, Eriksson P, Wennerholm UB, Dellborg M (2017). Effect of maternal age and cardiac disease severity on outcome of pregnancy in women with congenital heart disease.. Int J Cardiol.

[13] Duan R, Xu X, Wang X, Yu H, You Y, Liu X (2016). Pregnancy outcome in women with Eisenmenger's syndrome: a case series from west China.. BMC Pregnancy Childbirth..

[14] Slaibi A, Ibraheem B, Mohanna F (2021). Challenging management of a pregnancy complicated by Eisenmenger syndrome; A case report.. Ann Med Surg (Lond)..

[15] Yuan SM (2016). Eisenmenger Syndrome in Pregnancy.. Braz J Cardiovasc Surg..

